# Spectrum of central nervous system involvement in rheumatic diseases: pictorial essay

**DOI:** 10.1590/0100-3984.2016.0066

**Published:** 2018

**Authors:** Renata Mendes Vieira, Felipe Barjud Pereira do Nascimento, Alcino Alves Barbosa Júnior, Inês Carmelita Minniti Rodrigues Pereira, Zoraida Sachetto, Simone Appenzeller, Fabiano Reis

**Affiliations:** 1 MD, Resident in Radiology and Diagnostic Imaging at the Universidade Estadual de Campinas (Unicamp), Campinas, SP, Brazil.; 2 MD, Neuroradiologist at the Hospital Israelita Albert Einstein, São Paulo, SP, Brazil.; 3 Medical Coordinator of the Neuroradiology Group at the Hospital Israelita Albert Einstein, Professor of Neuromorphology at the Faculdade de Ciências da Saúde Albert Einstein, São Paulo, SP, Brazil.; 4 PhD, Professor in the Department of Radiology and Diagnostic Imaging of the Universidade Estadual de Campinas (Unicamp), Campinas, SP, Brazil.; 5 PhD, Professor in the Department of Internal Medicine of the Universidade Estadual de Campinas (Unicamp), Campinas, SP, Brazil.; 6 Associate Professor in the Department of Rheumatology of the Universidade Estadual de Campinas (Unicamp), Campinas, SP, Brazil.; 7 PhD, Head of the Neuroradiology Sector, Professor in the Department of Radiology and Diagnostic Imaging of the Universidade Estadual de Campinas (Unicamp), Campinas, SP, Brazil.

**Keywords:** Lupus erythematosus, systemic, Arthritis, rheumatoid, Behçet syndrome, Scleroderma, systemic, Spondylitis, ankylosing

## Abstract

The rheumatic diseases, which include systemic lupus erythematosus, rheumatoid arthritis, Behçet's disease, scleroderma, and ankylosing spondylitis, are characterized by involvement of connective tissue, with multiple manifestations. In those diseases, there can be involvement of the peripheral or central nervous system, and that involvement can be primary, presenting as a major feature of the clinical presentation, or secondary, as an effect of the drugs used in order to control a given disease or its complications. Knowledge of the wide variety of imaging findings is crucial to the diagnosis of a rheumatic disease, especially in the early stages, enabling effective treatment and minimizing disability. This pictorial essay, presenting cases from the records of two tertiary teaching hospitals, encompasses cases of patients diagnosed with rheumatic disease and illustrates the neuroradiological findings on magnetic resonance imaging and computed tomography, in order to emphasize the importance of these methods for properly diagnosing rheumatic diseases.

## INTRODUCTION

Rheumatic diseases, including systemic lupus erythematosus, rheumatoid arthritis, Behçet's disease, scleroderma, and ankylosing spondylitis, are characterized by involvement of the connective tissue of the entire body^(1-3)^. In such diseases, involvement of the central nervous system (CNS) or peripheral nervous system can be one of the main characteristics of the clinical picture or can be accompanied by other symptoms.

CNS involvement, be it primary or secondary, can occur in the course of rheumatic diseases. The diagnosis depends on the knowledge of a variety of imaging findings, allows effective treatment, and minimizes disability.

This work brings together case files obtained over the last 15 years from the Radiology Departments of the Hospital das Clínicas da Universidade Estadual de Campinas and the Hospital Israelita Albert Einstein. We included patients with a confirmed diagnosis of systemic lupus erythematosus, rheumatoid arthritis, Behçet's disease, scleroderma, or ankylosing spondylitis. The objective was to illustrate neuroradiological findings in magnetic resonance imaging (MRI) and computed tomography (CT), emphasizing the usefulness of both for diagnostic purposes. The study was approved by the research ethics committees of the two institutions.

## SYSTEMIC LUPUS ERYTHEMATOSUS

Systemic lupus erythematosus is an autoimmune disease that often involves the CNS, with variable incidence. Neuropsychiatric syndromes are divided into central neurological disorders (aseptic meningitis, cerebrovascular disease, demyelination, headache, benign intracranial hypertension, movement disorders, myelopathy, and epilepsy) and psychiatric disorders (acute confusional state, anxiety disorder, cognitive dysfunction, and affective disorders)^(4)^. Imaging, especially MRI of the skull, facilitates the diagnosis. MRI is more sensitive than is CT and is considered the gold standard for evaluation of disease progression, and even for investigation. However, there are no specific MRI findings, and in view of the broad spectrum of clinical, biochemical, and pathological manifestations, the radiological findings are pleomorphic^(4)^.

**Figure 1:**
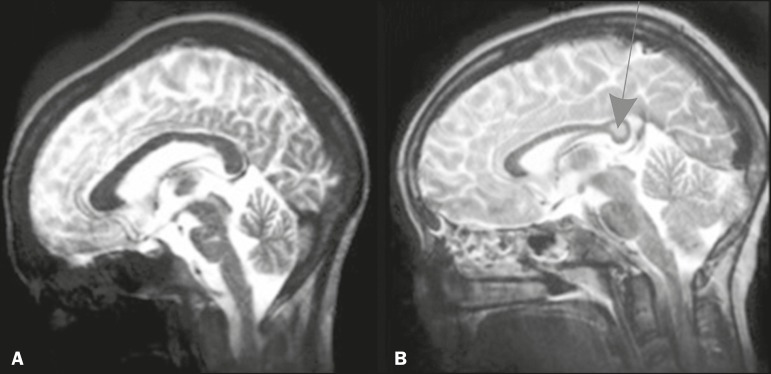
MRI scans of the skull in several patients diagnosed with systemic lupus erythematosus. **A:** Young female patient diagnosed with systemic lupus erythematosus seven years prior. Sagittal T2-weighted image showing a hyperintense linear lesion in the pons and signs of cerebellar (especially anterior lobe) and cerebral volume reduction. **B:** Adolescent female patient. Sagittal T2-weighted image showing a hyperintense lesion in the splenium of the corpus callosum (arrow).

Cerebral infarctions are common findings, not only in deep regions but also in cortical and subcortical regions. Regional or diffuse cerebral atrophy is common (Figure 1A). Transient, reversible focal lesions (which can be accompanied by vasogenic edema) may also be observed in the splenium of the corpus callosum (Figure 1B). Less commonly, venous sinus thrombosis can be seen, with or without other peripheral thrombotic events, especially in patients with antiphospholipid syndrome.

**Figure 2:**
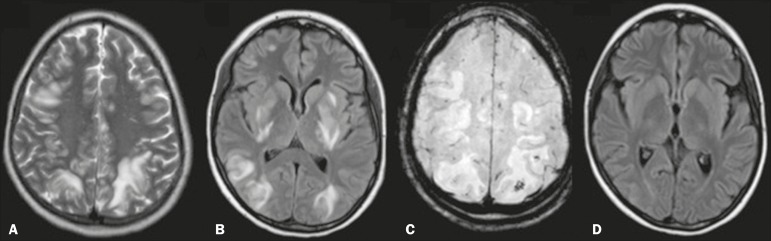
MRI scan of the skull of a female patient diagnosed with systemic lupus erythematosus who developed reversible posterior encephalopathy. **A,B:** Axial T2-weighted and FLAIR images, respectively, showing bilateral cortical-subcortical areas of hyperintense signals in the occipital, parietal, and frontal lobes, with a slight expansile effect, including the basal ganglia. **C:** Susceptibility-weighted imaging sequence identifying a subcortical focus with a hypointense signal in the left parietal lobe (petechial hemorrhage). **D:** Follow-up image obtained after the acute stage, showing reduction of the previously demonstrated lesions.

Reversible posterior encephalopathy is observed, particularly when there is use of a maintenance dose of corticosteroids and hypertensive crisis (Figure 2). It typically occurs in the parietal-occipital regions (the posterior circulation being most susceptible), although it can occur in the frontal region and basal nuclei, in which case it is accompanied by petechial hemorrhages.

In rare cases, bilateral, symmetric intraparenchymal calcifications are observed, especially in the basal nuclei, case reports citing a probable relationship with vasculopathy^(5)^. Another unusual form is a demyelinating disease, similar to multiple sclerosis, known as lupoid sclerosis. On MRI, there are findings of demyelinating lesions, including the multifocal white matter lesions seen in multiple sclerosis and vaso-occlusive disease, such as systemic lupus erythematosus.

Myelitis is one of the most debilitating complications, typically with an MRI pattern of transverse myelitis: a long segment of involvement (height greater than two to three vertebral bodies), involving both halves of the medulla, with a swelling effect^(6)^.

**Figure 3:**
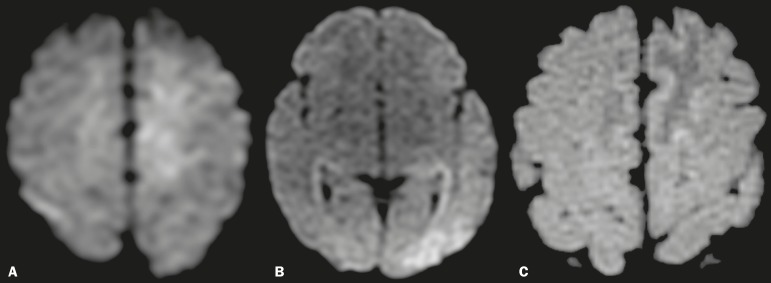
Diffusion-weighted MRI scans of the brain of a newborn, born to a woman with lupus, who presented a convulsive episode, showing areas of restricted diffusion in the upper left frontal gyrus and left temporo-occipital region (**A,B**), which showed a differential with changes related to status epilepticus and ischemic events related to neonatal lupus. A blood test revealed anti-Ro positivity. An MRI scan obtained one week later showed a zone of signal intensity change in the parasagittal frontoparietal region, consistent with ischemic lesions in the subacute phase (**C**).

Another spectrum is neonatal lupus with cardiac and cutaneous anomalies in newborns of mothers with anti-Ro/SSA and anti-La/SSB autoantibodies. In isolation, CNS involvement is rare and is described as transient vasculopathy. One case report in the literature described ischemic infarction secondary to CNS vasculitis^(7)^. In the present study, we describe the case of a neonate with convulsive seizures after birth and signs of acute ischemia on MRI (Figure 3).

## RHEUMATOID ARTHRITIS

**Figure 4:**
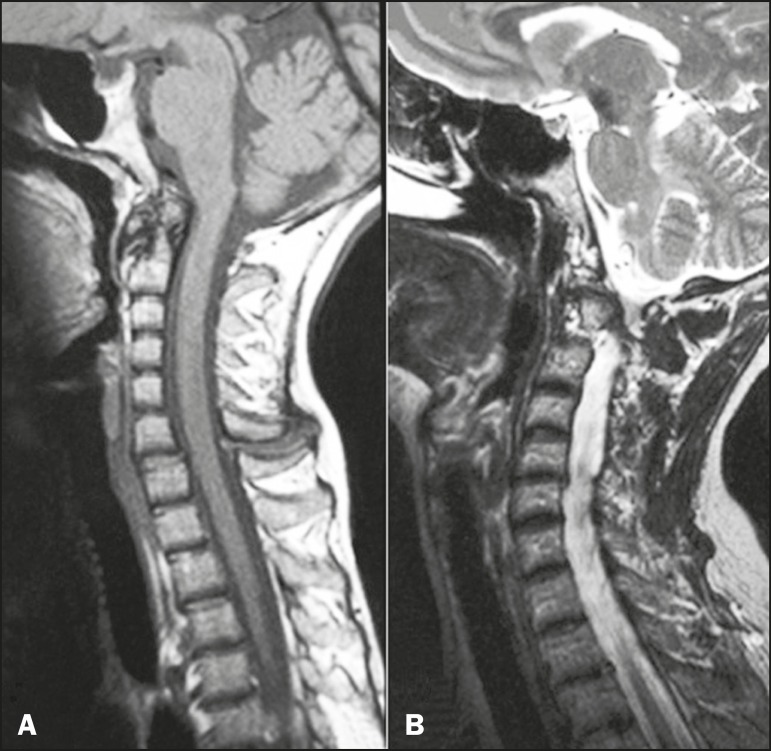
A female patient diagnosed with rheumatoid arthritis. T1-weighted and T-2 weighted MRI scans (**A** and **B**, respectively) of the cervical spine MRI images showing inflammatory synovitis with pannus formation between C1-C2, with erosive bone lesions, and basilar invagination.

Rheumatoid arthritis is the most common inflammatory disease involving the spine and has a predilection for the craniocervical junction. The three main manifestations in the cervical spine are basilar invagination, atlantoaxial instability, and subaxial subluxation. The main finding on MRI is pannus formation around the atlanto-odontoid joint, consisting of inflammatory proliferation of synovial tissue, with a hypointense signal in T1-weighted sequences and a hyperintense signal when there is a long repetition time, accompanied by odontoid erosions, with enhancement after administration of paramagnetic contrast. Subluxations, such as atlanto-occipital subluxation (5% of patients), can lead to spinal canal stenosis and compressive myelopathies^(8)^. In the present study, we illustrate a case of involvement of the atlanto-occipital joint (Figure 4). Another manifestation in the CNS is rheumatoid meningitis, with involvement of the meninges characterized by sulcal hyperintensity in fluid-attenuated inversion recovery (FLAIR) sequences, together with thickening and enhancement of the leptomeninges and pachymeninges. The diagnosis is confirmed through histopathological analysis, which will show rheumatoid nodules, nonspecific meningeal inflammation or vasculitis^(9)^.

## BEHÇET'S DISEASE

**Figure 5:**
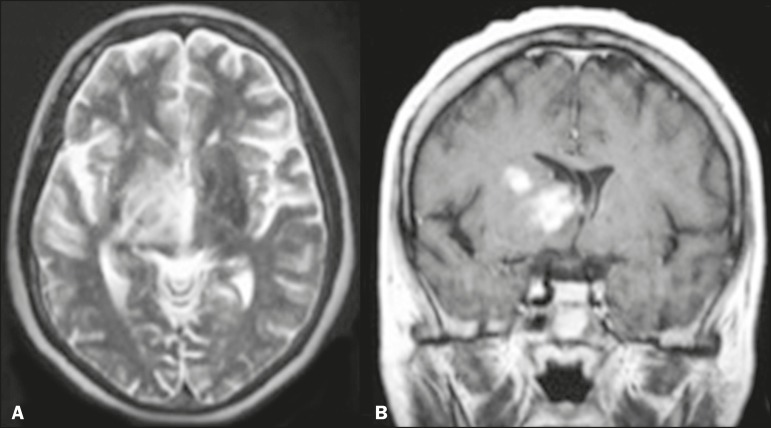
Female patient diagnosed with Behçet's disease. Contrast-enhanced T2-weighted and T1-weighted MRI scans of the skull showing a subcapsular lesion with an expansile effect in the right thalamus, extending to the subthalamus and right cerebral peduncle, with diffuse, heterogeneous contrast enhancement.

**Figure 6:**
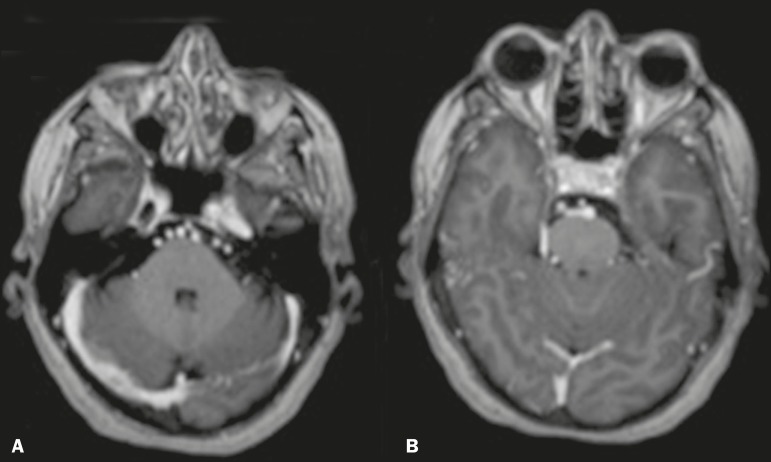
Female patient diagnosed with Behçet's disease. Contrast-enhanced T1-weighted MRI scan of the skull showing entanglement of vessels in the prepontine cistern supplied by dural branches of enlarged caliber, with a fistula to the right sigmoid sinus, consistent with dural fistula.

Behçet's disease has vascular, inflammatory, and multisystemic origins. Increasing clinical and imaging evidence suggests that the primary neurological involvement in Behçet's disease can be subclassified into several forms. The most common such form is characterized as an inflammatory vascular disease of the CNS with focal or multifocal involvement of the parenchyma, which in most patients presents as a subacute brainstem syndrome accompanied by hemiparesis. Another form, mildly symptomatic and with a better prognosis, can be caused by isolated cerebral venous thrombosis and intracranial hypertension. During the acute phase, Behçet's disease can present hyperintense lesions on contrast-enhanced T2-weighted/FLAIR MRI images. The subthalamic region and brainstem are common sites of involvement, although there can also be involvement of the basal nuclei, cerebral hemispheres, and spinal cord^(10)^. In the present study, we observed an atypical presentation: a pseudotumor of inflammatory origin caused by Behçet's disease (diagnosis confirmed by stereotactic biopsy with anatomical pathology of gliosis with gemistocytic astrocytes), as depicted in Figure 5. We also describe a patient who developed a dural fistula after venous thrombosis (Figure 6).

## SCLERODERMA

Scleroderma is a rare autoimmune disease characterized by inflammation, vascular injury, and fibrosis. Linear scleroderma includes a spectrum from localized scleroderma to systemic sclerosis.

**Figure 7:**
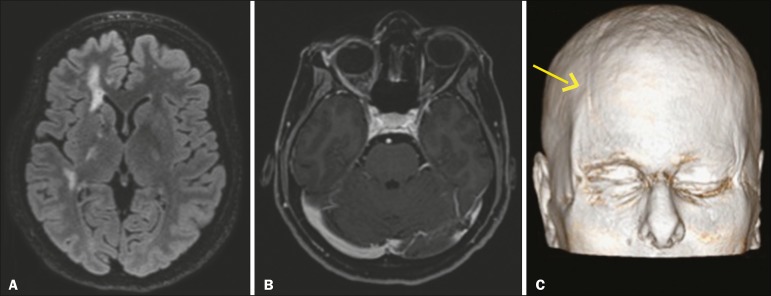
Female patient being followed for convulsions. **A:** Axial MRI FLAIR sequence of the skull showing areas of signal hyperintensity in the subcortical white matter and the internal capsule on the right. **B:** Contrast-enhanced T1-weighted sequence showing facial asymmetry, best demonstrated in three-dimensional reconstruction (arrow in **C**).

Linear scleroderma is considered a disease limited to the skin, subcutaneous tissue, and underlying bone. In the craniofacial subtype, there is neurological involvement. Linear scleroderma "en coup de sabre" is a rare subtype of linear scleroderma. In its typical presentation, it affects the frontoparietal region. The "en coup de sabre" lesion is defined as a banded lesion on the frontoparietal scalp and forehead. The associated atrophy of muscle structures, cartilage, and facial bones should raise the hypothesis of Parry-Romberg syndrome, up to 28% of patients with linear scleroderma manifest features of that syndrome, such as slowly progressive unilateral atrophy of the face. CT scans of the skull show narrowing of the external diploic space, cerebral atrophy, subcortical lesions, focal subcortical calcifications, and pachymeningeal abnormalities. Intraparenchymal calcifications involving basil nuclei, the thalamus, and dentate nuclei are more common ipsilateral to the cutaneous lesion, although contralateral involvement can occur. In T2-weighted MRI sequences, there are usually foci of hyperintense signals, mainly in the subcortical white matter but also in the corpus callosum, deep gray nuclei, and brainstem. Cerebral atrophy is subtle and focal, characterized by lack of definition of the cortical-subcortical interface, cortical thickening, and abnormal gyral pattern^(11,12)^. We illustrate a case of linear scleroderma "en coup de sabre" in combination with Parry-Romberg syndrome (Figure 7).

## ANKYLOSING SPONDYLITIS

**Figure 8:**
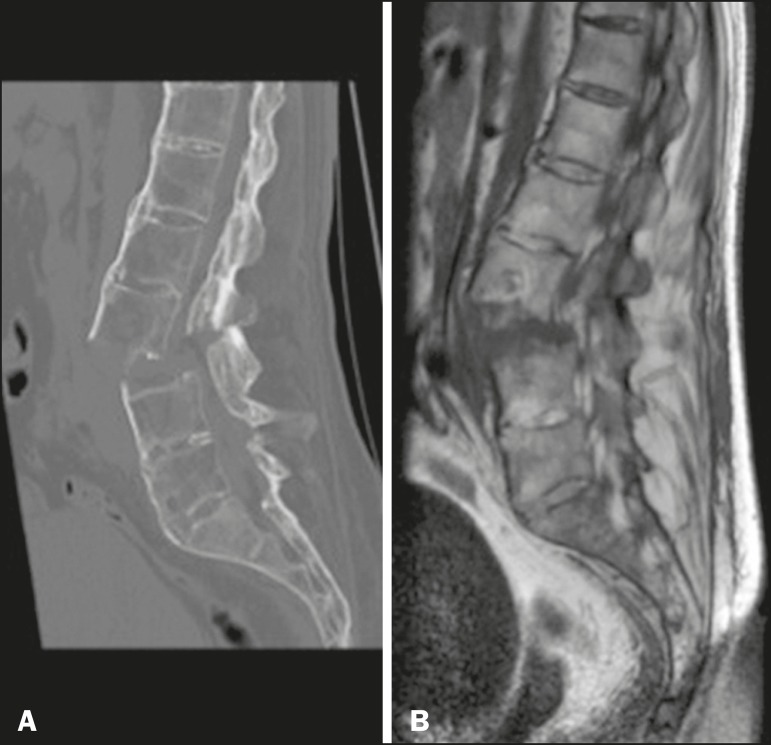
Male patient, with no previous diagnosis of ankylosing spondylitis, who presented to the emergency room with acute back pain. CT of the lumbar spine, with three-dimensional reconstructions, and MRI of the lumbar spine, showing fracture of elements of the three-column spine of Denis (anterior, middle, and posterior), characterizing an unstable fracture, a characteristic lesion of the disease.

Ankylosing spondylitis is an inflammatory arthropathy with enthesopathy of the axial skeleton and complications that affect the neuraxis. The most common symptoms are insidious lumbar pain, stiffness and asymmetric peripheral oligoarthritis. Osteoporosis is frequent and the risk of fractures is increased in relation to the general population. Characteristic imaging findings include diffuse osteopenia; bilateral, symmetric sacroiliitis; and calcification of the longitudinal ligaments, syndesmophytes forming the so-called "bamboo spine". Transverse fractures that cross the entire spine, associated with minor trauma, usually in the cervicothoracic or thoracolumbar junction, can occur, resulting in myelopathy and epidural hematoma. MRI shows Romanus and Anderson lesions, which are signal intensity changes in the margins and center of the vertebral bodies, respectively^(13)^. In the present study, we identified the case of a patient with transverse spine fracture in the lumbar spine after mild trauma, with no previous diagnosis of ankylosing spondylitis (Figure 8).

## CONCLUSION

CNS involvement in rheumatic diseases is pleomorphic and nonspecific. However, neuroimaging patterns can affect the diagnosis, in the initial manifestation and in the evaluation of complications.
